# Prostatic Hypertrophy1Read before the Gross Medical Club, May 31, 1901.

**Published:** 1901-08

**Authors:** Marshall Clinton

**Affiliations:** Buffalo, N.Y.; Attending surgeon, Erie County Hospital; assistant attending surgeon, Sisters’ Hospital and Buffalo Children’s Hospital; surgeon Pennsylvania R. R., etc.


### Prostatic Hypertrophy.[1]

BY MARSHALL CLINTON, M. D., Buffalo, N. Y.,

Attending surgeon, Erie County Hospital; assistant attending surgeon, Sisters' Hospital and Buffalo Children's Hospital; surgeon Pennsylvania R. R., etc.

THE object of this paper is to try and provoke a free discussion among the members and ascertain what ideas you have on the treatment and caring for patients of this kind, who present to you phases of this trouble that the hospital servitor does not see. You have the opportunity to watch patients from manhood to old age, to see a generation pass from the prime of life to senility, and among them must see prostatics who present many phases in the symptomatology of this complaint. What I particularly wish to find out from your experience is, how long a patient with an enlarged prostate will continue to use a catheter without developing a cystitis, and in cases in which it does develop how long before renal insufficiency asserts itself? How long after they present symptoms of a pyelonephritis do they sur-

1. Read before the Gross Medical Club, May 31, 1901.

vive? This last state is the one in which we generally see them in the hospital wards. In the writer's six years' hospital experience a good many cases have come under his observation, but no case has been under his care for six years. Hospital patients do not or can not give correct histories of these affections, even when they wish to; so it is to you we come to get an idea as to the length of time that usually elapses during the progress of the earlier stages. When, in your opinion, may we tell a patient that an operation is imperative; when, after signs of bladder weakness, will obstruction come on; and when after that will they have retention? This is one stage of the trouble when our patients present themselves, and are promptly scared away by the announcement that unless they submit to an operation they will get worse and die miserably.

The classification given us by Thompson of four divisions of chronic prostatic enlargement, is the one generally accepted by the authorities. To the practitioner, however, it matters little what type the chronic enlargement is; the symptom-pictures the patients present from the various types are the same. Probably no one ailment in all general surgery has so many sufferers between the ages of 50 and 75 as chronic prostatic hypertrophy. Unfortunately, the general surgeon sees these patients at a time when surgical skill will do little else than render easy a measured life, or push over the border line the tottering prostatic patient.

The symptomatology is familiar to us all. The forceless stream of urine, the dribbling at the end, the loss of that younger day bladder strength. Then the symptoms of beginning irritability at the neck of the bladder from congestion and later frequent urination from pouching at the neck and retention of part of the contents after each act. These symptoms become aggravated at night and the frequent urination is the symptom that usually points the patient's feet towards the physician's office. This symptom of frequent urination is due to a high specific gravity urine with a plus acid reaction being allowed to fall on an irritable prostatic urethra by the relaxed bladder neck during sleep.

With increasing enlargement comes difficulty in starting the stream. This symptom often portends more serious structural change in the prostate than frequent urination. In these cases where a large share of the irritable symptoms are left out there is a passive dilatation of the bladder wall and a constant backward pressure on the kidneys that results in kidney lesions out of all proportions to the symptoms, as compared with other irritable cases.

Residual urine, whether small or great in amount, is of no importance, as it is simply the measure of the bladder capacity until it becomes refilled or a cystitis develops. Complete retention is rarely an early symptom, usually occurring late in the disease after some sexual, gastronomic, or climateric disturbance.

Diagnosis is so generally clear from the history and age of the patient, that we will usually complete it with a simple rectal palpation, which method tells us more than any other or all others.

*Treatment.*—Medically speaking, prostatic hypertrophy is as hopeless to treat as fibroid uterus, but there are drugs that may be used when the hypertrophy has produced symptoms of cystitis, and the like. The medical treatment of this condition is safe only when the patient is under the care of a physician who is on the alert for the early signs of an incompetent kidney, or the results of back pressure or infection. Many cases fortunately do go through this period with a mild degree of prostatic hypertrophy and discomfort without anything more than a careful watching and an occasional instrumental dilatation. Many a man has lost his life who has drifted along with an enlarged prostate without the careful supervision necessary to prolong his life until structural changes have taken place in his kidneys, from which he cannot recover and from which he dies.

Palliative measures consist in the use of the catheter, steel sounds, and deep urethral dilators. When the resistance to a catheter is slight, the density of the prostate through the rectal wall is moderate, amount of residual urine small, then dilatation will be of great benefit. Dilatation should be persisted in to the largest sized sound that can enter the membraneous urethra. The constant use of the catheter is the commonest palliative measure and the most popular one up to the present. The dangers are sometimes misunderstood, and we hear this mode of procedure condemned when a large number of cases go through the prostatic age with very little inconvenience. If the catheter is to be used at all, its use should be begun early in the affection before any hyperemic changes have taken place in the urethra and bladder.

An uninjured urethra and healthy bladder wall is not a good medium for the growth of pathogenic bacteria, and a reasonable amount of cleanliness will avoid a cystitis. A patient should not be permitted to use the catheter until the bladder and urethra has reached a normal state of resisting power. The rule in catheterising of once a day for three ounces of residual urine, and once for every additional two ounces is a good one. Continuous drainage by retained catheter is recommended by some and is a valuable palliative measure in cases of acute cystitis engrafted on an old atonic condition of the bladder.

In all our patients when catheterisation is easy, the amount of residual urine small, when there is no cystitis, and the obstruction is moderate, no operation should be thought of. All prostatics do not need operation and many of them go through this period of their lives with very little trouble. What we need, then, is some definite rule which will guide us in advising operation. Structural changes in the prostate per se are not enough to make operation imperative. Even with a very much hypertrophied middle lobe cystitis per se is not enough to require operation. Both together do not always give us the required warning. The way in which these patients die is either from uremia or sepsis. Sepsis from bladder and subsequent kidney infection or from a nephritis due to infection or back pressure on the ureters. To the urinary secretion, then, must we look as the imperative guide as to whether an operation is indicated or not. Given a case of progressive prostatic hypertrophy with cystitis amenable to treatment and no involvement of the kidneys, our advice would be, not to operate. Given the same case, however, with signs of an albuminuria, which is persistent and has casts in some profusion scattered through it, and our advice will be always to operate.

The terrible mortality of the earlier and some later operations devised to relieve this condition cannot be laid at the door of the operations themselves, but to the fact that operations are too often undertaken when the patient's condition precludes the possibility of a recovery.

How often have we seen a septic, uremic soaked, half dead patient sent into the hospital to be operated on for this condition? And how utterly hopeless these cases are. Many if not all of these poor patients could be spared to helpful useful lives if only at some past date proper advice of their attending physician had been carried out. Under ordinary precautions the model operation done on a man with healthy, properly functionating kidneys, should not have any mortality. When, however, any operation requiring an anesthetic is attempted on a patient in the former condition, we can readily see why we have such a frightful mortality. At the first sign of beginning kidney degeneration—operate!

There have been a great many different methods of attempting radical cures of this affection and they all have their adherents. The literature on this subject is so vast that we will not attempt to review any but a small part of the various methods that have been suggested for the relief of this condition. Castration and vasectomy have not proven as efficacious as they were originally intended, and are only applicable in cases where there is no projection of the middle lobe of the prostate, the glands soft in feel, and the etiological factor largely sexual in its production. It may be tried in the earlier stage of prostatic enlargement for it should do no harm. Ligation of the internal iliacs is certainly as formidable an operation as prostatectomy. Bottini's operation has been abandoned by some operators as unsurgical and unsatisfactory. It is only suitable in a limited class of cases and is not one we would care to have as the radical operation. Prostatotomy by Bottini's method or any other is not a radical procedure though it was intended for such. Too large a per cent. are not cured—42.5 per cent. in 229 cases. Prostatotomy was the first of the operative procedures for the permanent relief of this condition, and is the parent of the modern and model operation—prostatectomy. Suprapubic cystotomy has been done a great deal, particularly for those cases associated with a great amount of cystitis, but is only a temporary operation at the best. I was forced to do one for a patient who was dying last summer from retention, as it was the only surgical procedure the relatives of the patient would consent to. He was not a case for operation and it was done to make his end easy and not with the idea of curing his pyelonephritis, which was killing him. It succeeded in as far as giving an outlet for the urine was concerned and was done under cocaine anesthesia. Could this patient have been given the benefit of an earlier operation he should have been alive and comfortable today.

Prostatectomy, or enucleation of the prostate is the model operation and has several minor variations for its accomplishment.

Parker Syms proposes to do a laparatomy, then a perineal cystotomy, and pushing the prostate and bladder into the perineal wound, enucleate without opening the bladder. Alexander's method is to keep outside the general peritoneal cavity with the suprapubic incision and carry it into the bladder. Then a grooved staff is inserted into the bladder in the urethra and an incision carried in the middle line back to the apex of the prostate and into the membranous urethra. With the left hand in the bladder above and pressing the prostate into the perineal wound, the prostate is enucleated by the first finger of the right hand.

This has not been found a difficult procedure by the various operators who have been prevailed upon to try it, and I saw a large prostate with an enormous middle lobe enucleated in seven minutes by Dr. Paul Thorndyke, of Boston. This operation is still too new to have a very large series of cases on which to base statistics, but the twenty odd reported by Alexander show a lower death-rate and a higher per cent of complete cures than any other method so far. This seems reasonable when we consider it is the only procedure which completely removes the offending member.

Mansell Moullin says:

> Thanks to the progress which has been made in the surgery of the prostate in the last few years, progress which only became possible when the true sexual character of the gland had been once more established, there is now no case of enlargement in which perfect relief cannot be obtained, provided only the secondary consequences which so often and so unnecessarily follow it, and which are due in the vast majority of cases to the careless use of catheters, have not allowed to work irreparable harm upon the walls of the bladder. In no department of surgery are the disastrous results of temporising and delay shown more clearly than in that which is concerned in the prostate.

We all realise the frequent difficulty of demonstrating to the patient the necessity of any operation, but if when a patient is carefully shown just what changes are going on, and what the results will be, if he is not under careful control, I feel sure that our operations will have a lower mortality, give more perfect results alike to the patient, physician, and surgeon, and save many a valued member of our communities to a helpful, useful, contented, old age.

466 FRANKLIN STREET.

---

INSOMNIA OF ALCOHOLISM.—Dr. D. R. Brown (*Trans. Seventh International Congress on the Abuse of Alcohol*) recommends for patients suffering with acute alcoholic delirium or alcoholic mania:

℞ Chloralamid. . . . . . . . . . 1.0 (gr. xv.)
Hyoscin hydrobrom . . . . . . . 0.0006 (gr. $\frac{1}{100}$.)

